# *Vitis vinifera* L. Diversity for Cations and Acidity Is Suitable for Breeding Fruits Coping With Climate Warming

**DOI:** 10.3389/fpls.2020.01175

**Published:** 2020-09-18

**Authors:** Antoine Bigard, Charles Romieu, Yannick Sire, Laurent Torregrosa

**Affiliations:** ^1^ AGAP, University of Montpellier, CIRAD, INRAe, Institut Agro, Montpellier, France; ^2^ UE INRAe de Pech Rouge, University of Montpellier, INRAe, Gruissan, France; ^3^ GENOVIGNE, University of Montpellier, IFV, INRAe, Institut Agro, Montpellier, France

**Keywords:** fleshy fruit, grape, cations, acidity, fruit quality, climate changes

## Abstract

The selection of grapevine varieties is considered to be the smartest strategy for adapting the viticulture to climate warming. Present knowledge of the diversity of grape solutes known to be influenced by temperature is too limited to perform genetic improvement strategies. This study aimed to characterize the diversity for major cations (K^+^, Mg^2+^, Ca^2+^, NH_4_^+^) of the *Vitis vinifera* fruit and their effect on acidity. Two developmental stages were targeted: the end of green growth, when organic acids reach a maximum, and the physiological ripe stage defined by the stopping of solutes and water import at the maximum volume of the berry. Twelve varieties and 21 microvines from the same segregating population were selected from preliminary phenotyping. The concentration of cations depended on the stage of fruit development, the genotype and the environment with GxE effects. In the ripe grape, K^+^ concentration varied from 28 to 57 mmol.L^-1^ with other cations being less concentrated. Combined with the variation in organic acids, cation concentration diversity resulted in titratable acidity of the ripe fruit ranging from 38 to 215 meq.L^-1^. These results open new perspectives for the selection of varieties to mitigate the adverse effects of climate warming on grape quality.

## Introduction

With a world production ranging from 75 to 85 million tons a year, grapes are one of the most commonly eaten fleshy fruits. At a global level, viticulture is mainly dedicated to table grape production (www.oiv.int, www.fao.org), but the production of juice, dried grapes or wines can also be important to local economies. For instance, wine production in France represents an annual economic balance of more than 11 billion € (www.franceagrimer.fr).

The grape is mainly composed of water (75-85% of the fresh weight), sugars (10-15%), organic acids (0.2-1%), minerals (0.1-0.5%), polyphenolic compounds (0.1-0.2%) and aroma compounds (<0.1%). The development of the berry involves two growth periods (Mullins et al., 1992). The first phase results from cell division and a first run of vacuolar expansion due to the accumulation of organic acids (Kliewer, 1965; Ojeda et al., 1999). During this phase, inorganic compounds are accumulated, e.g. Ca^2+^ with a central role in cell wall structure, but also K^+^, NH_4_^+^, and Mg^2+^ as counter-ions for vacuolar anions (Doneche and Chardonnay, 1992; Mpelasoka et al., 2003; Bashir and Kaur, 2018). After a lag phase, berries soften, phloem unloading shifts from the symplasmic to the apoplasmic pathway (Zhang et al., 2006), triggering the sudden acceleration of sugar import and a second phase of water import, known as ripening (Matthews et al., 1987). The organization of the sugar import pathway remains uncertain and it was proposed that it could be energized by the discharge of a phloem potassium battery (Nieves-Cordones et al., 2020).

Cations participate in many aspects of fruit development through the regulation of various metabolic pathways (Ramesh-Kumar et al., 2006; Maathuis, 2009; Song et al., 2018). For instance, K^+^ regulates almost 60 enzymes, including protein synthesis, oxidative metabolism and photosynthesis (Vicente et al., 2009). Magnesium is a major constituent of the chlorophyll and serves important biochemical functions in protein synthesis. It is also involved in the regulation of energetic metabolism as a constituent of the Mg-ATP or Mg-ADP complex and in the regulation of the Calvin cycle (Vicente et al., 2009). In plants, Ca^2+^ is primarily associated with the cell wall pectin materials with a role in turgor regulation associated with organ rheological properties. It is also a mediator of plant responses, such as abiotic stress signaling. Potassium is the predominant cation in plants (Doneche and Chardonnay, 1992; Bonomelli and Ruiz, 2010; Bashir and Kaur, 2018). Given their sequential accumulation, organic acids (up to 250 mmol.L^-1^) and sugars (up to 1 mol.L^-1^) are the main contributors of the osmotic potential during, respectively, green stage and ripening, far above inorganic compounds (Storey, 1987). Indeed, K^+^ remains below 100 mmol.L^-1^, and both magnesium and calcium remain under 5 mmol.L^-1^ all throughout grapevine fruit development.

Grape juice acidity is dependent on cations which neutralize and precipitate a fraction of organic acids (Champagnol, 1984). The balance between acidity and sugars is known to be a major determinant of wine organoleptic quality (La Rosa, 1955; Jaime-Baro, 1973; Kourakou, 1974; Du Plessis, 1984). Climate changes have already impacted vine development and grape composition (Ojeda et al, 2017; Drappier et al., 2017). The impact of environmental factors on grapevine reproductive development and on the accumulation of primary metabolites has been extensively described (Butrose, 1969a; Butrose, 1969b; Webb et al., 2007; Dai et al., 2011; Greer, 2012; Xu et al., 2014; Rienth et al., 2016; Luchaire et al., 2017; Torregrosa et al., 2017). However, the impact of environmental factors on cation accumulation has received little attention. A panel of viticultural practices can be implemented to modify the balance between primary metabolites and cations, but these practices have been shown to induce deleterious effects on plant development or secondary metabolite accumulation (Champagnol, 1984; Greer et al., 2011; Bobeica et al., 2015). Similarly, post-harvest corrections of the acido-basic balance can improve (most often in increasing) acidity, from organic acid additon to cation removal through electrodialysis (Escudier et al., 2012; Sweetman et al., 2014).

The use of genetic diversity and breeding appear as smart options for selecting genotypes better adapted to global warming (Ollat et al., 2015; Gascuel et al., 2017; Torregrosa et al., 2017). However, modern viticulture only uses a limited fraction of the potential diversity (Wolkovich et al., 2018). For instance, the 30 first varieties propagated in France accounted for 85% of all plants produced in 2017 (www.franceagrimer.fr). There are several studies on the diversity for fruit size, primary and secondary metabolites in *V. vinifera* (Boursiquot et al., 1995; Shiraishi et al., 2010; Houel et al., 2013; Preiner et al., 2013; Teixeira et al., 2013; Yinshan et al., 2017) or in segregating progenies (Doligez et al., 2006; Liu et al., 2006; Liu et al., 2007; Mejia et al., 2007; Duchêne et al., 2012; Doligez et al., 2013; Duchêne et al., 2013; Chen et al., 2015; Costantini et al., 2015; Houel et al., 2015). However, while there are some reports about the capacity of the rootstock to modulate K^+^ and Mg^2+^ scion nutritional status (Kodur et al., 2009), little is known about the genetic diversity of cation composition of the grapevine fruit.

Recently, Bigard et al. (2019) pointed out the impossibility to unambiguously define developmental stages in unsynchronized berry populations. Attempts were made to compare genotypes more precisely by sorting berries at precise physiological stages, i.e. the onset, and the arrest, of phloem unloading through the apoplasmic pathway (Bigard et al., 2018). Using the same approaches, we have characterized the diversity for the major cation concentration (i.e. Potassium, Calcium, Magnesium and Ammonium) in grapevine fruit and the resulting acidity.

## Materials and Methods

### Plant Material and Growing Conditions

Berries from two *Vitis vinifera* subsets (**Table S1**) were analysed. The first subset included: i) in 2016, 12 ungrafted accessions established on non-irrigated sandy soils at the Grapevine Biological Resources Centre of Vassal (*www6.montpellier.inrae.fr/vassal*) among which ii) 6 genotypes were re-phenotyped in 2017 at the experimental vineyard of Institut Agro of Montpellier (*en.montpellier-supagro.fr/research/experimental-research-platforms/pierre-galet-experimental-vineyard*), all grafted on SO4 rootstock, established on gravelly soils. Five to twenty replicated plants were available each year, managed by spur pruning, vertical shoot positioning (VSP) and fertirrigation. The number of clusters was reduced to 4-8 per vine by cluster thinning to avoid source/sink unbalance effects.

The second subset included a progeny of 21 microvines derived from a cross between the Picovine 00C001V0008 (*Vvgai1*/*Vvgai1*) bearing the *Dwarf and Rapid Cycling and Flowering* (DRCF) trait (Chaib et al., 2010) and the Ugni Blanc fleshless berry mutant (Fernandez et al., 2006). These microvine plants were grown in pot (3-6 years old) in semi-controlled conditions at the INRA experimental centre of Pech-Rouge (France) in 2016. In 2017, 6 of the 21 microvines were re-phenotyped at the Montpellier SupAgro campus. Lateral branches were systematically removed to standardize vegetative and reproductive development, maintaining a single proleptic shoot per plant as described in Luchaire et al. (2017) and Torregrosa et al. (2019).

Both subsets of genotypes were exposed to different growing conditions to assess GxE effects: microvines were maintained at 15/25 +/- 3°C night/day temperatures and watered at full PET (potential evapotranspiration) in greenhouse while macrovines were grown outdoors for 2 years in 2 environments differing for soils and climate conditions (**Table S2**). In the manuscript, the terms experiments, environment, or year are indifferently used to represent the variations due to environment *sensu lato* (E).

### Fruit Sampling Methods

Samples at key stages of berry development were obtained as described in Bigard et al. (2018). For varieties, in 2016, individual berry softening was monitored by hand on 9 pre-selected bunches. When the first soft berries were detected, it was assumed that all the remaining ones had reached the green lag-phase, and 4-30 hard berries were sampled. Then, 3, 4 and 5 weeks later, 2-54 berries were sampled on the same bunches. Unfortunately, all berries were already soft at the beginning of the experiment on Trousseau, so the green stage is missing for this genotype. From the 3 dates of sampling, only the samples displaying the maximum average berry volume were analyzed for cations. In 2017, the same sampling methodology was repeated at the green stage. Berry growth was then non-destructively monitored upon immersing in water two reference clusters per genotype 3 times a week (Torregrosa et al., 2008). Triplicates (3 x 30 berries) were sampled at 3-day intervals when berry growth started to slow down. In 2017, samples for green stages of Trousseau and Muscat d’Alexandrie were lost.

For microvines, plants were grown until displaying all reproductive stages from flowering to berry shriveling (Torregrosa et al., 2019). In 2016 and 2017, 2-11 green lag-phase berries were sampled per cluster as described above for macrovines. Regarding ripe stage, in 2016, 2-13 berries were collected from 3 successive bunches above the one exhibiting the first signs of shrivelling. In 2017, 5-8 berries were sampled on clusters 3 to 5 levels below the bunch showing berry softening.

### Sample Preparation

For both genotype subsets, samples displaying the maximum average berry volume were selected for cation analyses. In 2016, berries were ground with a mortar and pestle at room temperature and frozen at -30°C. Before analyses, samples were first heated at 60°C for 30 min. Crude samples were then vigorously vortexed for 30 seconds and centrifuged 5 min at 18,500 g at 20°C. Clear supernatant was 10X-diluted with 0.2 N HCl, and then filtered on 0.2 µm cellulose acetate filters and finally analyzed. In 2017, after weighing, berries were immersed in 4 volumes of 0.25 N HCl, de-seeded and incubated for 2 days at room temperature. After a gentle shaking, supernatants were 10X-diluted with water and stored at -30°C. Then, for analysis, samples were defrosted at room temperature and vigorously shaken.

### Cations Analysis and Titratable Acidity Calculation

Samples were centrifuged for 3 min at 12000 rpm (20°C) and then 10 µl clear supernatant was directly injected *via* a Waters^®^ 717 (Waters, www.waters.com) device for HPLC through a Waters^®^ IC-Pak Cation M/D 3.9x150 mm column (20°C) eluted at 1ml/min flow rate with 0.004 N HNO_3_ as mobile phase. Then, K^+^, Ca^2+^, Mg^2+^ and NH_4_^+^ concentrations were measured using a Shimadzu^®^ CDD-10A conductometer (Shimadzu, www.shimadzu.fr) and the Waters^®^ EMPOWER-3 peak integration software. Recalculated titratable acidity (RTA) was expressed in meq.L^-1^, as the sum of malic and tartaric acids (Bigard et al., 2018) minus K^+^, these elements being respectively the main anions and the major cation (Boulton, 1980).

### Data Analysis and Graphic Representations

Statistical analysis was conducted using R-software version 3.4.3 (R Core Team, 2017). Raw data and R codes will be provided upon request. Statistical analysis of G, E and GxE interactions were based on genotypes repeated in 2016 and 2017 and determined using an ANOVA II test for parametric data subsets and a Two-way ordinal regression test for non-parametric data subsets. Correlations were calculated using a Pearson’s correlation coefficient test.

## Results and Discussion

### The Complexity of the Sampling Strategy

**Figure 1:**
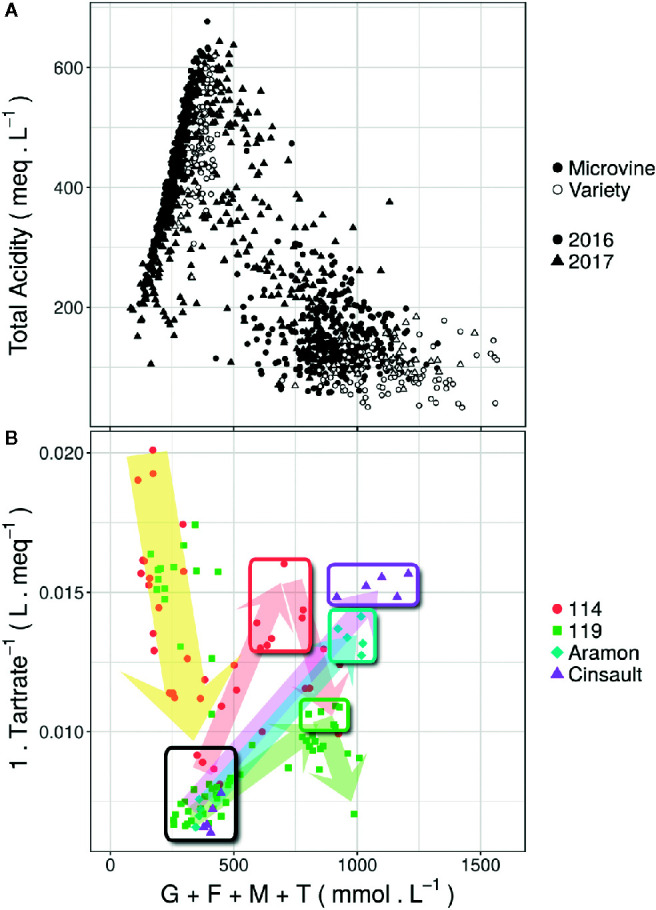
Evolution of the total acidity **(A)** and fruit growth **(B)**. **(A)** shows the level of acidity in all collected samples as a function of the sum of major osmotica (Glucose + Fructose + Malate + Tartrate). **(B)** shows the volume of the fruits for the varieties Aramon and Cinsault, and the microvine 114 and 119 fruit growth expressed as 1/tartaric acid concentration (Bigard et al., 2019). Black square shows the position of green stage samples. Colored squares represent the position of the samples selected to represent physiological ripe stage.

The changes in total acidity (**Figure 1A**), berry growth (**Figure 1B**) and malic acid (**Figure 3** in Bigard et al., 2018) with respect to the accumulation of major osmotica are proxies to assess the advancement of fruit ripening. For both genotype subsets (**Figure 1A**), berry total acidity falls in the expected order of magnitude. The developmental stage of the different samples can be outlined using berry growth (**Figure 1B**), which is correlated to the dilution of the tartaric acid (Bigard et al., 2019). For microvines, samples are distributed according to the classical development program of the grapevine fruit from green to over-ripe stages. The selection performed on the base of berry volume allowed for the refining of targeted stages (**Figure 1B**), the berry volume virtually doubling between green and ripe stage, as widely accepted for most *V. vinifera* varieties (Houel et al., 2013; Bigard et al., 2018).

For all species forming clusters of small fruits such as redcurrant, blueberry, blackcurrant, dates, coffee or grapevine, the phenotyping is complicated by the heterogeneity and asynchrony of single fruit development (Lobos and Hancock, 2015; Lobos et al., 2017). A cluster is composed of berries at different developmental stages (Coombe, 1992; Shahood, 2017; Bigard et al., 2019; Shahood et al., 2019). In genetic and most physiological studies, a grape phenological stage corresponds to a mix of berries representing the heterogeneity of the fruits at plot level (Preiner et al., 2013; Houel et al., 2015; Yinshan et al., 2017; Duchêne et al., 2020). Obviously, the concept of developmental stage remains equivocal in such heterogeneous samples. Indeed, a phenological stage must be defined by intrinsic physiological parameters, such as the onset and the arrest of sugar loading, which may occur at different harvest dates or brix, according to the genotype (Bigard et al., 2019; Shahood et al., 2019).

The sampling procedure implemented here aimed to obtain samples representative of the onset and at the arrest of the second growth period which must be clearly distinguished from the following shriveling phase. Our approach includes two intrinsic limitations. Firstly, the sampling of the last hard berries to represent the green stage and the collection of spatial (microvines, Luchaire et al., 2017) or temporal (varieties) series of berries to select the ripe stage, both presume that all fruits are synchronized. Secondly, a sample averaging unsynchronized berries can only provide an approximation of the maximum concentration reached by a berry (Bigard et al., 2019). Despite these limitations, the interpretation of **Figures 1A, B**, and the **Figure 3** of Bigard et al. (2018), suggests that proposed methods can reduce some usual averaging artifacts in genotypic diversity studies.

### Potassium

**Figure 2:**
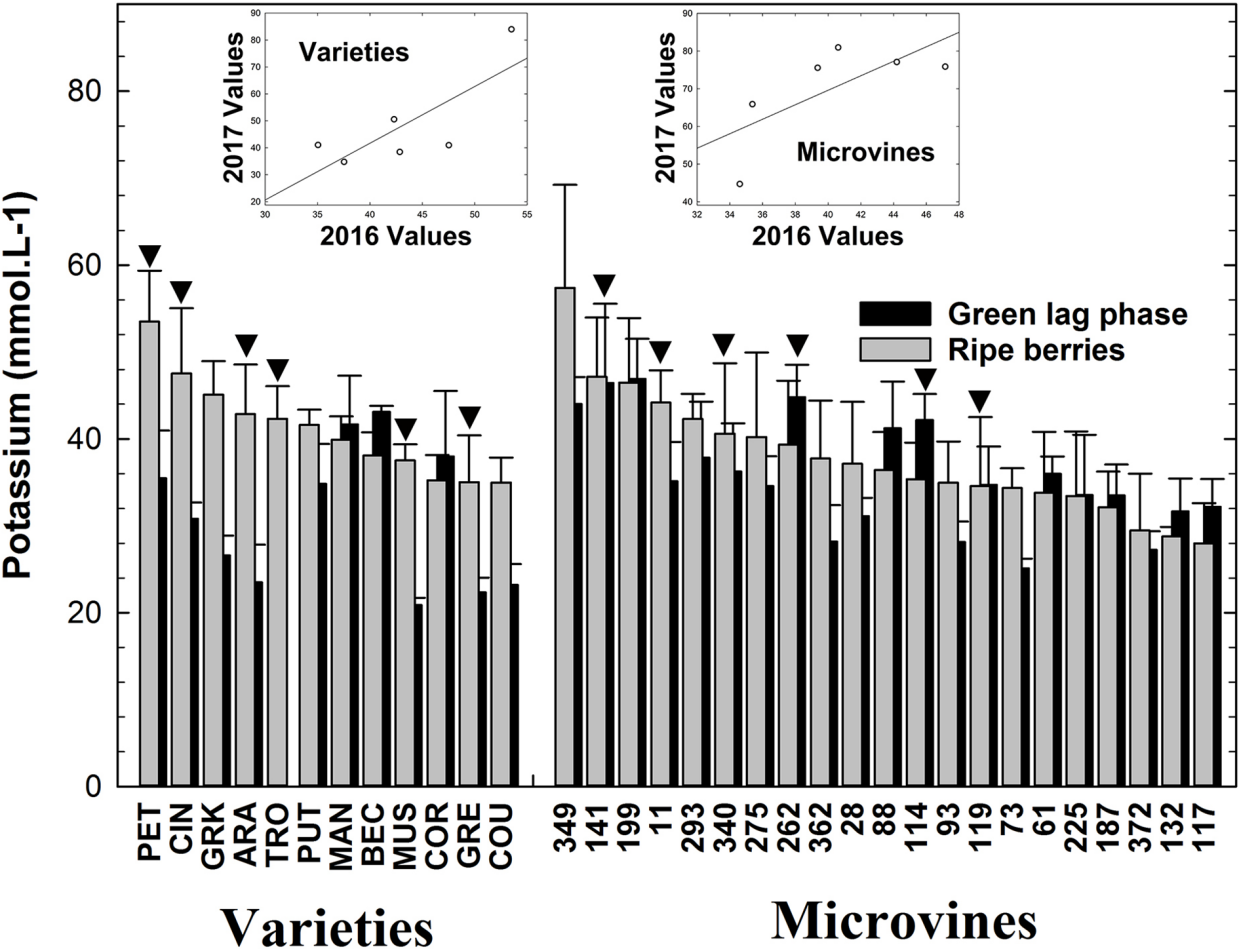
K^+^ concentrations in the grapevine fruit at the end of green growth and at physiological ripe stage. Bar chart represents 2016 mean values with the corresponding SE. Genotypes experimented in 2016 and 2017 are indicated by a black down-pointing triangle. Inserted plots show the relationships between the mean values of both years (see **Tables S3**, **S4** for detailed numeric values and statistics).

Varieties and microvines displayed very limited changes in K^+^ concentration during ripening (**Figure S5**). Since the volume of the *V. vinifera* berry doubles during ripening (Houel et al., 2013; Bigard et al., 2018), this implies a very faint K^+^ accumulation rate, with respect to major organic osmotica. For varieties which were grown outdoors, K^+^ concentrations ranged from 21 (Muscat d’Alexandrie) to 43 (Béclan) mmol.L^-1^ at green stage and from 35 (Couston) to 54 (Petit Manseng) mmol.L^-1^ at ripe stage (**Figure 2**) with very little evolution during ripening for most varieties (**Figure S6**). Microvines grown in greenhouse displayed similar K^+^ concentrations, ranging from 25 (microvines n°73) to 47 (microvine n°199) mmol.L^-1^ at green stage and from 28 (microvine n° 117) to 57 (microvine n°349) mmol.L^-1^ at ripe stage with slight evolution during ripening for most lines (**Figure S7**). At the green stage, statistical analyses (**Tables S3**, **S4**) showed an effect of G, E and GxE on K^+^ accumulation for both varieties and microvines. At the ripe stage, G and GxE effects were statistically significant in varieties with no E effect. The strong G effect compared to the small GxE effect suggests a genetic control of this trait at the ripe stage in varieties. In the microvine subset, both G and E effects were statistically significant without interaction showing the strong genetic control of this trait inside a progeny.

Potassium concentrations observed here are either higher (Mpelasoka et al., 2003) or lower (Storey, 1987; Rogiers et al., 2017) than previously reported in other GxE backgrounds. It is known that agronomic factors (type of soil, fertilization, irrigation…) modulate K^+^ soil availability and influence its accumulation in grapes (Champagnol, 1984). Temperature during ripening can also modulate K^+^ concentration in ripe grapes (Mira de Orduña, 2010). Moreover, methodological factors can also make the comparison difficult. Indeed, around 50% of berry K^+^ is located in the skin, the extractability of which critically relies on the protocol, and may dramatically increase during ripening, due to marked modification in cell wall structure and tightness (Possner and Kliewer, 1985). Furthermore, K^+^ is prone to precipitation as potassium bitartrate in the juice, as the pH increases during ripening (Rienth et al., 2016). Hence the quantification of this element is dependent on the care taken to avoid its precipitation prior to analysis. In this study, sample preparation performed at high temperatures or in acid conditions and high dilution levels limited the precipitation of K^+^. However, as the first steps of sample preparation were different in 2016 and 2017, it cannot be excluded that extraction efficiency varied between the 2 years. Nevertheless, even this potentially limits the discussion on causality of year-to-year variations, Dumas et al. (2020) showed that the methods of extraction have little impact on genotypic comparisons.

Considering the diversity of genotypes and environments of this study, the variability for K^+^ content in the ripe fruit is quite moderate, suggesting a strong homeostasis for this element. There have been significant advances in the understanding of the mechanisms of K^+^ transport and accumulation in the last 10 years (Rogiers et al., 2017; Villete et al., 2020). Compared to other reports, our data reveals 2 interesting biological features: i) the low increase of K^+^ concentration during ripening and ii) the lack of environmental effect, when compared to sugars (Bigard et al., 2018). Differences with the previous reports could result from the sampling strategies. Generally, the determination of the ripe stage is based on unsorted berries harvested at technological maturity, after solutes and water importation stops. Here, we have taken care to assess solute concentrations before shriveling, that concentrates all the solutes of the berry, while their transport is definitively stopped.

### Magnesium

**Figure 3:**
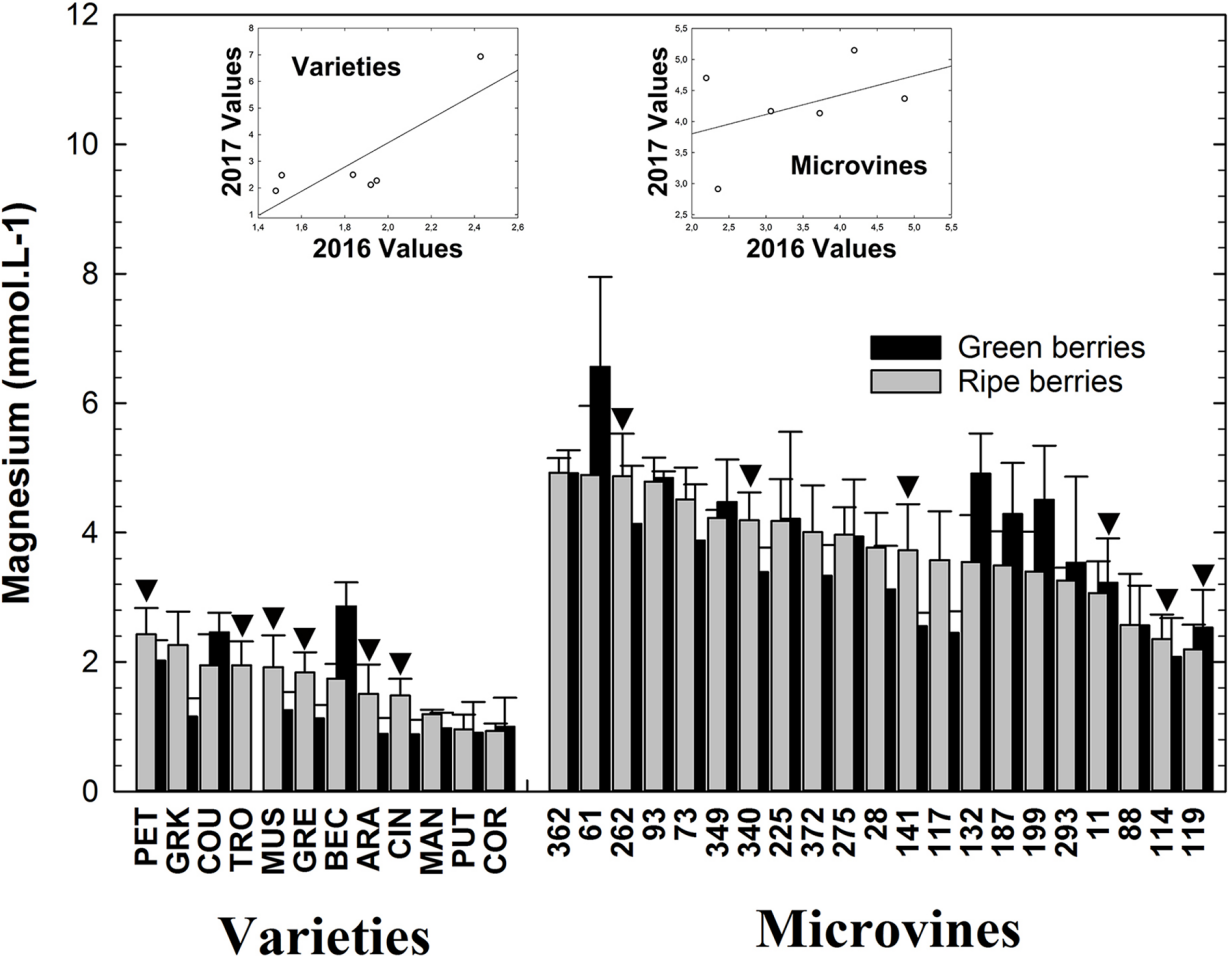
Mg^2+^ concentrations of the grapevine fruit at the end of green growth and at physiological ripe stage. Bar chart represents 2016 mean values with the corresponding SE. Genotypes experimented in 2016 and 2017 are indicated by a black down-pointing triangle. Inserted plots show the relationships between the mean values of both years (see **Tables S3**, **S4** for detailed numeric values and statistics).

Magnesium was less accumulated than potassium in all genotypes. At both fruit developmental stages, varieties accumulated less Mg^2+^ than microvines (**Figure S8**). At the green stage, Mg^2+^ ranged from 0.9 (Cinsaut) to 2.9 (Béclan) mmol.L^-1^ for varieties and from 2.1 (microvine n° 114) to 6.6 (microvine n°61) mmol.L^-1^ for microvines (**Figure 3**). At the ripe stage, values ranged from 0.9 (Cornifesto) to 2.4 (Petit Manseng) mmol.L^-1^ for varieties and from 2.2 (microvine n°119) to 4.9 (microvine n°362) mmol.L^-1^ for microvines. At the green stage, statistical analyses (**Tables S3**, **S4**) showed an effect of G, E and GxE for both varieties and microvines, with a lower significance for E effects in microvines. At the ripe stage, statistical analyses showed a significant effect of E, G without GxE interaction for varieties, and G, E and GxE effects for microvines. As for K^+^, Mg^2+^ concentration evolved little during the ripening growth period (**Figures S9**, **S10**). In this study, Mg^2+^ concentrations were found higher in the microvine progeny than in varieties, which exhibited similar values than previously reported (Mpelasoka et al., 2003). This could be due to the specific condition of cultivation of microvines which were grown in a greenhouse and on their own roots.

### Calcium

**Figure 4:**
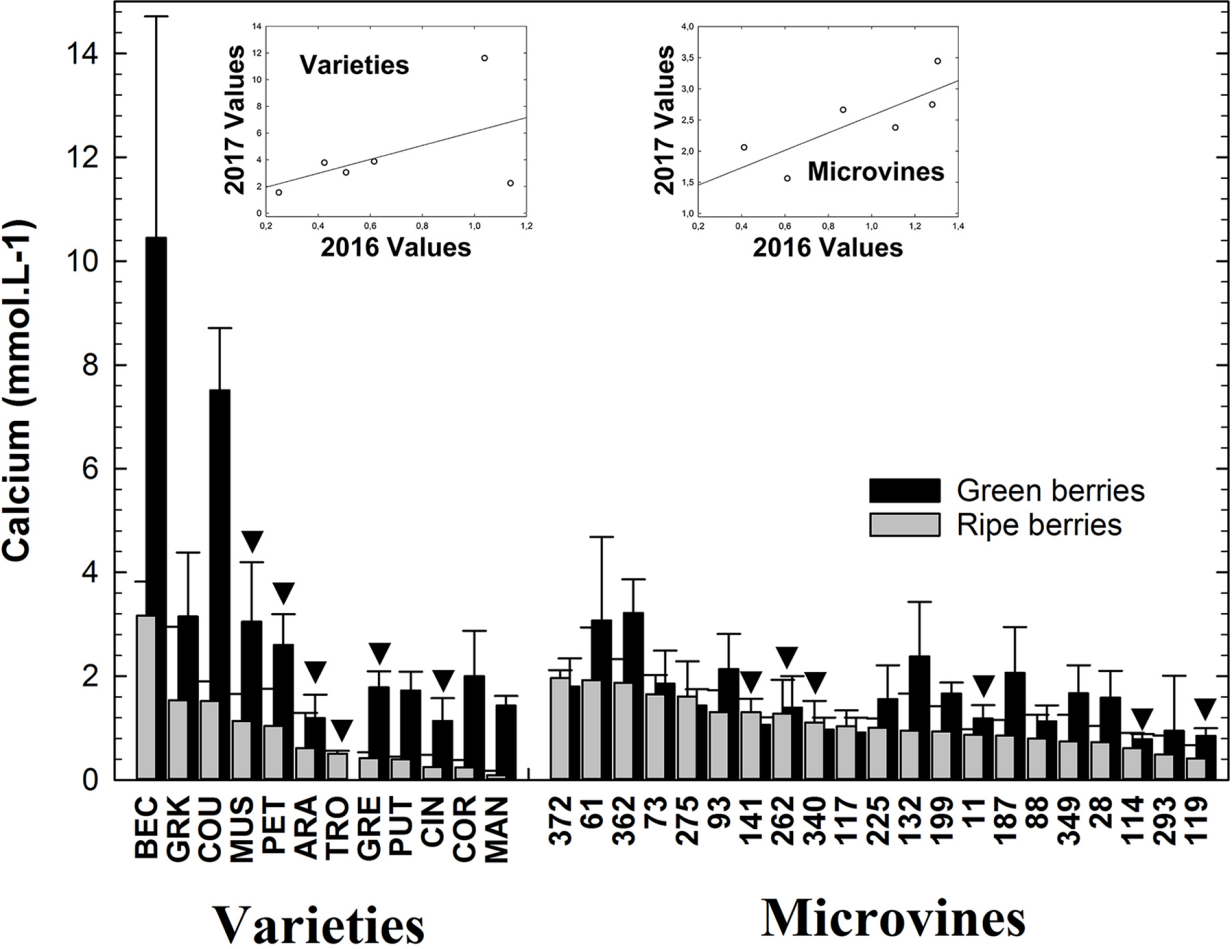
Ca^2+^ concentrations in the grapevine fruit at the end of green growth and at physiological ripe stage. Bar chart represents 2016 mean values with the corresponding SE. Genotypes experimented in 2016 and 2017 are indicated by a black down-pointing triangle. Inserted plots show the relationships between the mean values of both years (see **Tables S3**, **S4** for detailed numeric values and statistics).

This element is accumulated at a much lower rate than K^+^ and Mg^2+^ in grapes (**Figure 4**). Varieties and microvines tended to display a similar range of Ca^2+^ concentrations values either in green or ripe fruits (**Figure S11**). At the green stage, concentrations in Ca^2+^ ranged from 1.1 (Cinsaut) to 10.5 (Béclan) mmol.L^-1^ for varieties and from 0.8 (microvine n°114) to 3.2 (microvine n°362) mmol.L^-1^ for microvines. At the ripe stage, values ranged from 0.1 (Mandilaria) to 3.2 (Béclan) mmol.L^-1^ for varieties and from 0.4 (microvine n°119) to 2 (microvine n°372) mmol.L^-1^ for microvines. The concentrations in Ca^2+^ tended to decrease in macrovine and microvines (**Figures S12**, **S13**). At the green stage, statistical analyses (**Tables S3**, **S4**) showed an effect of G, E and GxE for both varieties and microvines. At the ripe stage. statistical analyses showed a significant effect of E, G and GxE interaction for varieties, and G, E without GxE interaction for microvines also suggesting a possible control of this trait during breeding.

In this study, Ca^2+^ content in green and ripe berries was found higher than previously reported (Mpelasoka et al., 2003; Bonomelli and Ruiz, 2010; Bashir and Kaur, 2018). Interestingly, conversely to K^+^ and Mg^2+^, on both subsets, the concentration in Ca^2+^ decreased significantly during ripening while the volume of the berry usually doubled (Bigard et al., 2018). Due to a poor mobility of Ca^2+^ in the phloem (Hocking et al., 2016), this element is mainly accumulated during green berry growth to support cell division and structure. During grape ripening, which is associated with a second phase of growth by vacuolar expansion (Dai et al., 2011), this element is being diluted.

### Ammonium

**Figure 5:**
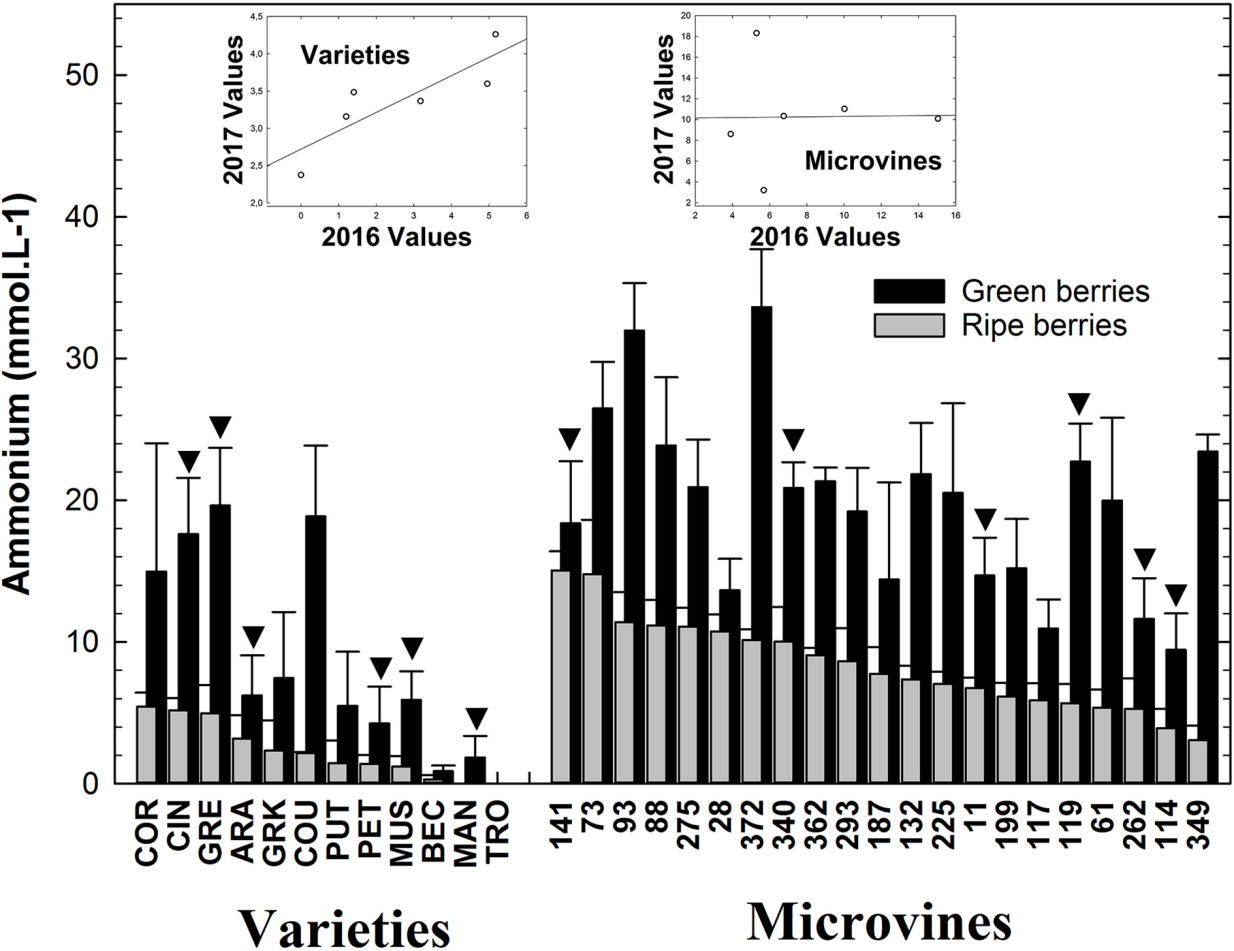
NH_4_^+^ concentrations in the grapevine fruit at the end of green growth and at physiological ripe stage. Bar chart represents 2016 mean values with the corresponding SE. Genotypes experimented in 2016 and 2017 are indicated by a black down-pointing triangle. Inserted plots show the relationships between the mean values of both years (see **Tables S3**, **S4** for detailed numeric values and statistics).

Varieties and microvines displayed a similar range of NH_4_^+^ concentrations values either in green or ripe fruits (**Figure S14**) with a clear tendency to decrease for both subset during ripening (**Figures S15**, **S16**). For varieties, NH_4_^+^ concentrations ranged from 0.9 (Béclan) to 19.6 (Grenache) mmol.L^-1^ at green stage and from 0 (Mandilaria) to 5.5 (Cornifesto) mmol.L^-1^ at ripe stage (**Figure 5**). In microvines, NH_4_^+^ ranged from 9.5 (microvine n°114) to 33.7 (microvine n°372) mmol.L^-1^ at green stage and from 3 (microvine n°349) to 15 (microvines n°141) mmol.L^-1^ at ripe stage. Statistical analyses (**Table S4**) showed an effect of G and GxE on NH_4_^+^ at green stage for the microvine line. Only microvine 114 kept high NH_4_^+^ concentrations for both years. For varieties (**Table S3**), they showed an effect of G, E and GxE. At the ripe stage, statistical analyses showed a significant effect of E, G with GxE interaction for both varieties and microvines. Grape displays the same range of NH_4_^+^ concentrations as other fleshy fruits such as red fleshy fruits, e.g. Strawberry (Taghavi et al., 2004), Blackberry or Raspberry (Strik and Bryla, 2015).

Ammonium is a source of yeast assimilable nitrogen (YAN) conditioning grape juice fermentation. Its accumulation is dependent on agronomical (cover grass, yield, fertilization) and environmental factors making the comparison between data obtained in different experimental contexts difficult. In this study, despite some effects of the year and the interaction GxE, we have observed that a fraction of the phenotypic diversity for this trait is genotype-dependent. In some genotypes, the amounts accumulated during berry development (up to 15 mmol.L^-1^) potentially cover yeast needs while in some varieties the level of NH_4_^+^ could be limiting for wine processing (Salmon, 1996; Taillandier et al., 2007).

### Recalculated Titratable Acidity (RTA)

**Figure 6:**
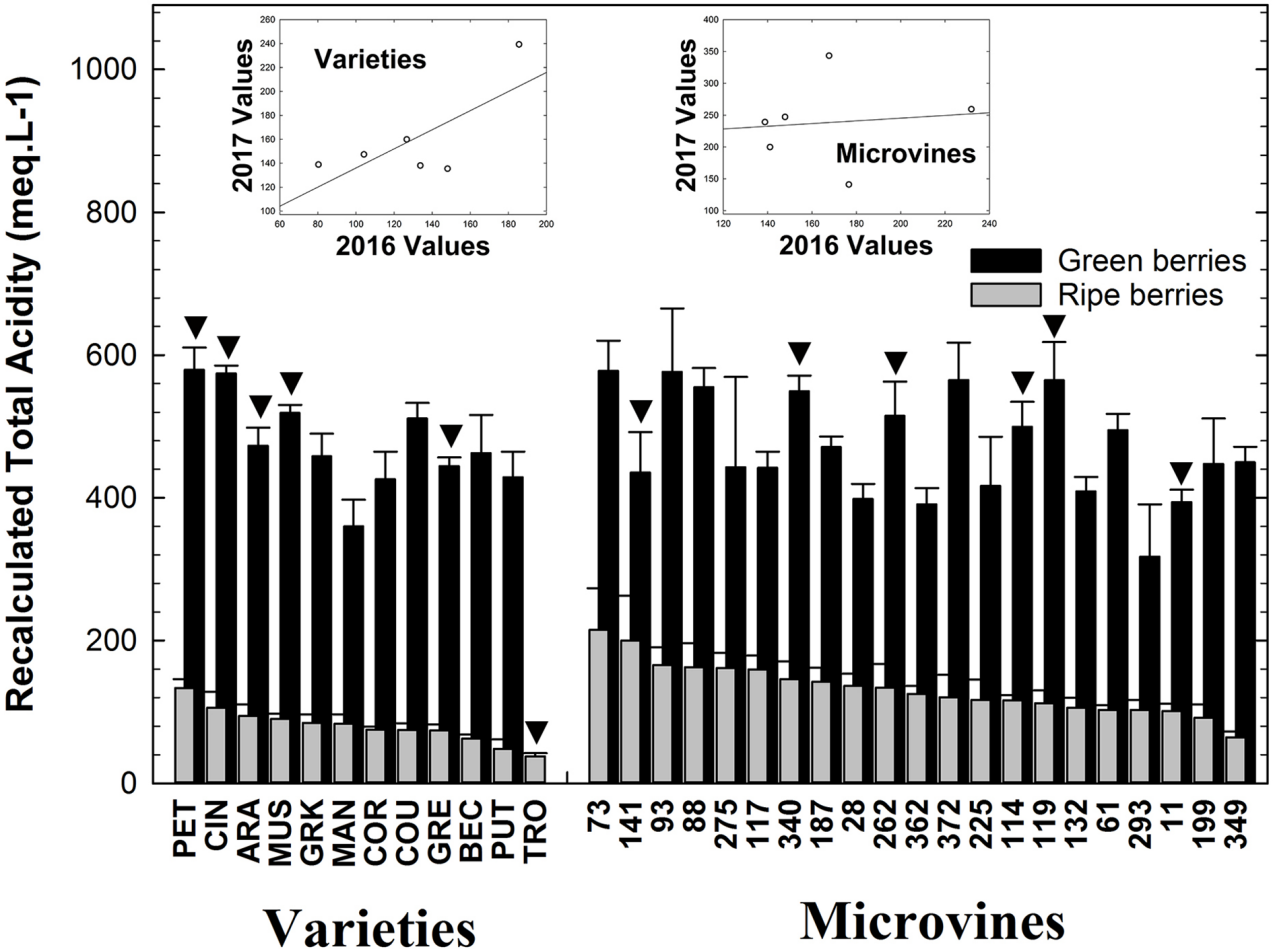
Recalculated Total Acidity (meq.L^-1^) the grapevine fruit at the end of green growth and at physiological ripe stage. Bar chart represents 2016 mean values with the corresponding SE. Genotypes experimented in 2016 and 2017 are indicated by a black down-pointing triangle. Inserted plots show the relationships between the mean values of both years (see **Tables S3**, **S4** for detailed numeric values and statistics).

As largely documented in grapevine, the acidity significantly decreased during ripening for both subsets (**Figures S17**-**S19**). Recalculated titratable acidity at the green stage ranged from 360 (Mandilaria) to 580 (Petit Manseng) meq.L^-1^ for varieties and from 318 (microvine n°293) to 578 (microvine n°73) meq.L^-1^ for microvines (**Figure 6**). At the physiological ripe stage, RTA varied from 38 (Trousseau) to 134 (Petit Manseng) meq.L^-1^ for varieties and from 64 (microvine n°349) to 215 (microvine n°73) meq.L^-1^ for microvines. At the green stage, statistical analyses (**Tables S3**, **S4**) showed an effect of G, E and GxE for varieties and a statistically significant effect on G with no E effect and no interactions for microvines. At the ripe stage, statistical analyses showed a significant effect of E, G with GxE interaction for both varieties and microvines. Those results are concomitant with the E, G and GxE effects obtained in Bigard et al. (2018) at the ripe stage for malate + tartrate concentration, showing the difficulty of findinding a genetic control of this trait.

Grape acidity is a major challenge in viticulture (Champagnol, 1984; Sweetman et al., 2014; Ollat et al., 2018). The effect of temperature on grape aciditity is well documented (Kliewer and Lider, 1970; Butrose et al., 1971; Seguin et al., 2004; Rienth et al., 2016). Following the report of Bigard et al. (2018) that presented the genetic diversity for anions (i.e. organic acids), here we analyzed the cation variations providing an overview of the diversity of the main determinants of the acidity of the grapes.

### Correlations Between Traits

For microvines at the green stage, Mg^2+^ was correlated with Ca^2+^ in 2016 (0.73, *p*-Value < 0.05) and NH_4_^+^ with the malic/tartaric acid ratio in 2017 (0.66, *p*-Value < 0.05). Several significant correlations appeared at ripe stage during both years: between Ca^2+^ and K^+^ (0.77, *p*-Value < 0.05), between RTA and NH_4_^+^ (0.73, *p*-Value < 0.05), and between RTA and K^+^ (0.62, p-Value < 0.05). Glucose was also correlated with Mg^2+^ both years (0.66 and 0.75, *p*-Value < 0.05), malic acid with NH_4_^+^ in 2016 (0.71, *p*-Value < 0.05). For varieties, at green stage in 2016, Ca^2+^ was correlated to Mg^2+^ (0.79, *p*-Value < 0.05) and tartaric acid to Mg^2+^ (0.65, *p*-Value < 0.05). At the ripe stage, very strong correlations were found in both years: between Ca^2+^ and Mg^2+^ (0.96, *p*-Value < 0.05), between K^+^ and Mg^2+^ (0.86, *p*-Value < 0.05), between K^+^ and Ca^2+^ (0.81, *p*-Value < 0.05). In 2016, glucose was correlated to Mg^2+^ (0.70, *p*-Value < 0.05). In 2017, K^+^ was correlated to berry weight (-0.61, *p*-Value < 0.05) and tartaric acid was correlated to K^+^ (0.71, *p*-Value < 0.05), to Ca^2+^ (0.79, *p*-Value < 0.05) and to Mg^2+^ (0.72, *p*-Value < 0.05). All cations analysed except NH_4_^+^, had an absolute correlation higher than 0.60 with RTA (p-Value < 0.05).

In this study, the strong impact of the environment was clearly visible as values were higher in 2016 than 2017 for cations accumulation. The highest correlations were found between Mg^2+^ and Ca^2+^ in varieties, both cations being known to have similar patterns of accumulation as expected from their common transport by xylem, and their absence from phloem sap (Glad et al., 1992a; Glad et al., 1992b).

In the range of the genotypes studied here, while the level of sugar concentrations increased by a factor 10 during ripening (Bigard et al., 2018), only a weak increase of K^+^ concentration was observed. In this respect, the Petit Manseng, a variety which accumulates a huge amount of sugars in the fruit (Bigard et al., 2018) only exhibited little variations of K^+^ concentration during ripening. This observation doesn’t support the hypothesis of an interdependent import of sugars and K^+^ during ripening (Coetzee et al., 2019; Duchêne et al., 2020; Nieves-Cordones et al., 2020). Because the berries shrivel after the arrest of phloem unloading (Du Plessis, 1984; Bigard et al., 2019), an important part of the co-variations of sugars and K+ concentration during ripening could be linked to water loss. Hence the importance of clearly defining the stages of sampling so as not to confuse the import and the concentration of fruit solutes.

### Cation and Acidity Diversity in a Breeding Perspective

**Figure 7:**
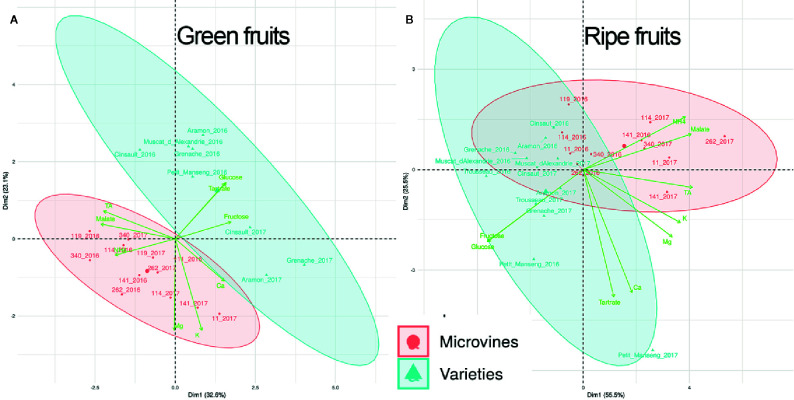
Principal component analyses of all variables collected for variety and microvine subsets at green **(A)** and physiological ripe **(B)** stages: K^+^, Ca^2+^, Mg^2+^, NH_4_^+^, RTA (recalculated titratable acidity), combined with variables reported in Bigard et al. (2018), i.e. Glucose, Fructose, Tartrate (tartaric acid), Malate (malic acid) concentrations.

The variables collected in this study at green and ripe stages for 12 accessions repeated in 2016 and 2017 (K^+^, Mg^2+^, Ca^2+^, NH_4_^+^, Titratable acidity) with the data of primary metabolites (Glucose, Fructose, Tartaric and Malic acids) from Bigard et al. (2018) were submitted to a PCA (**Figure 7**). At the green stage, PCA (**Figure 7A**) represents 55.7% of the variability observable. This analysis shows that varieties accumulated more sugars than microvines, with less NH_4_^+^. The total acidity (but not Tartaric acid) and cations (except NH_4_^+^) appear to be genotype-dependent in both genotype subsets. At the ripe stage, PCA (**Figure 7B**) represents 81% of the variability observable, which is higher than the green stage PCA. Both sugars were well correlated, as reported in Bigard et al. (2018). Another interesting observation is the link between malic acid and NH_4_^+^, which was already mentioned in the correlation section, in particular at the ripe stage. It is empirically known in viticulture (Champagnol, 1984) that soil and fertilization management or the use of vigorous rootstocks, can increase the content in nitrogen of the grapes and also increase malic acid accumulation, as the results of complex interactions between plant vigor and microclimate.

To establish a selection strategy, it is critical to assess the genetic potential of the targeted species (Lobos et al., 2017; Torregrosa et al., 2017). This includes the estimation of the available genetic diversity and the possibility to segregate targeted traits. For grapevine, as for other fruit crops, both the nutritional and the organoleptic components need to be considered. Indeed, in countries where nutrition is not secured either in quantity or quality, it may be critical for breeding fruit crops to focus on nutrient concentrations (Luby, 2009). This is particularly true for the regions where fresh grapes or derived products (juice, raisins) are a significant part of the human diet, such as Asia or South America (www.OIV.int). For fruits and non-fermented derivative products, vitamins and antioxidant compounds but also sugars and minerals are important nutritional components (Simon, 2014).

The amount of sugar at the ripe stage in grapes at physiological ripe stage can vary from 0.8 to 1.4 mol.L^-1^ depending on the genotype (Bigard et al., 2018), which represents a significant potential of calories. The dietary reference index for K^+^ is 120 mmol.day^-1^ (IOM, 2004). Potassium is the predominant cation of most fleshy fruits where it can be accumulated up to 75 mmol.Kg^-1^ in Banana for instance (Wall, 2006) and up to 60 mmol.Kg^-1^ in grapes (this study). Magnesium is the second abundant cation in fleshy fruits (Wall, 2006), with a dietary reference index of 13 mmol.day^-1^ (IOM, 2000). In some genotypes, we have shown here that Mg^2+^ can be accumulated up to 5 mmol.Kg^-1^ at physiological ripe stage. So table grape and derivative products (juice or raisins), can be a source of energy, K^+^ and Mg^2+^ for human nutrition. For Ca^2+^, in regards to the level of the dietary reference index (250 mmol.day^-1^, IOM, 2004) and considering the concentrations observed here (less than 3 mmol.Kg^-1^ at physiological ripe stage), grape consumption in fresh or as juice can only be a minor contributor to the human diet.

Regarding organoleptic properties, either for fresh grapes or derivative products (juice or wine), one important parameter to be discussed in light of our data is the sugar/acidity balance (Boulton, 1980; Du Plessis, 1984). Breeding programs are ongoing in Brazil and in France to select grape juice or wine varieties with improved sugar/acidity balance (Ritschel et al., 2014; Escudier et al., 2016). Due to several mechanisms, climate warming tends to increase the concentration in sugars in the grape at ripe stage (Ollat et al., 2018; Arrizabagala et al., 2018; Torregrosa et al., 2017) while decreasing the acidity (Lakso and Kliewer, 1978; Romieu et al., 2018; Van Leeuwen et al., 2019). In this study, PCA analysis showed that sugars and K^+^ are orthogonal at both developmental stages (**Figure 7**). The lack of univocal relationship between sugar and K^+^ accumulation during the phloem unloading period was recently reported by Bigard et al. (2019). This result is now confirmed from a genetic point of view. Consequently, it seems rather unlikely that the selection of low K^+^ accumulator genotypes would help in reducing the excessive sugar concentrations triggered by global warming, as proposed by Duchêne et al. (2020).

In Bigard et al. (2018), we have shown that either sugar or organic acid concentration can be independently selected in existing variety germplasms or in segregating populations. Here, we showed that cation concentrations display a significant genetic diversity and that this parameter can provide additional phenotypic diversity for the acidity of the grape. This diversity would be an effective alternative to physical or chemical methods currently allowed to improve grape must or juice acidity (Escudier et al., 2012; Sweetman et al., 2014). Indeed, while European regulations (EEC-606/2009) limit the addition of organic acids from 20-33 meq.L^-1^ depending on the product and the region, the OIV codex determines a maximum of 54 meq.L^-1^ (OENO 3/99 and OENO 13/01) for these corrections. The correction of cation concentrations by ion exchange resins or by bipolar membrane electrodialysis is also limited to 54 meq.L^-1^ (CEE 53/2011, OENO 360/2010) for conventional wines. Electro-membrane based and chemical processes cannot be combined and no correction is allowed for the production of organic wines.

The data reported here in complement to the report of Bigard et al. (2018), shows that a significant genotypic diversity is prevalent in *V. vinifera* for fruit composition at physiological ripe stage. This study also shows that parameters determining berry growth, organic and inorganic solute accumulation, i.e. sugar loading, organic acid synthesis and dilution, and major cation importation can be manipulated by crossbreeding. This opens interesting perspectives for the selection of grape varieties displaying specific fruit composition traits, with some of them being potentially useful to mitigate some adverse effects of climate warming on grape quality.

## Data Availability Statement

All datasets presented in this study are included in the article/**Supplementary Material**.

## Author Contributions

LT, CR, and AB designed the experiments. AB, YS, and CR performed the experiments. AB, LT, and CR drafted and edited the manuscript. All authors contributed to the article and approved the submitted version.

## Funding

This work was supported by the CIVB (Comité Interprofessionnel des Vins de Bordeaux), the Jean Poupelain foundation, the government of Occitanie, INRAe and the Institut Agro de Montpellier.

## Conflict of Interest

The authors declare that the research was conducted in the absence of any commercial or financial relationships that could be construed as a potential conflict of interest.

## Supplementary Material

Supplementary Table 1 List of the genotypes of the 2 subsets of genotypes.

Supplementary Table 2 Sum of the GDD (growing degree days) in base 10 and means of the average of the maximum temperatures during the 4 months of sampling outdoors (varieties) or in greenhouse (microvines).

Supplementary Table 3 Mean values and statistics of the fruit parameters measured for the 6 V. vinifera varieties in 2016 and 2017 at green and physiological fruit ripe stage.

Supplementary Table 4 Mean values and statistics of the fruit parameters measured for the 6 V. vinifera microvines in 2016 and 2017 at green and physiological fruit ripe stage.

Supplementary Figure 5 Potassium concentration as a function of sum of major osmotica (glucose + fructose + malate + tartrate) during ripening for all samples of this study **(S5)** and in variety **(S6)** and microvine **(S7)** subsets. Colored arrows show the evolution of K+ concentration for 3 genotypes of each subset.

Supplementary Figure 6 Magnesium concentrations as a function of sum of major osmotica (glucose + fructose + malate + tartrate) during ripening for all samples of this study **(S8)** and in variety **(S9)** and microvine **(S10)** subsets. Colored arrows show the evolution of Mg2^+^ concentration for 3 genotypes of each subset.

Supplementary Figure 7 Calcium concentrations as a function of sum of major osmotica (glucose + fructose + malate + tartrate) during ripening for all samples of this study **(S11)** and in variety **(S12)** and microvine **(S13)** subsets. Colored arrows show the evolution of Ca2^+^ concentration for 3 genotypes of each subset.

Supplementary Figure 8 Ammonium concentrations as a function of sum of major osmotica (glucose + fructose + malate + tartrate) during ripening for all samples of this study **(S14)** and in variety **(S15)** and microvine **(S16)** subsets. Colored arrows show the evolution of NH4^+^ concentration for 3 genotypes of each subset.

Supplementary Figure 9 Total acidity as a function of sum of major osmotica (glucose + fructose + malate + tartrate) during ripening for all samples of this study **(S17)** and in variety **(S18)** and microvine **(S19)** subsets. Colored arrows show the evolution of the total acidity for 3 genotypes of each subset.
