# Supplementary figures and images for: Vitis vinifera L. Diversity for Cations and Acidity Is Suitable for Breeding Fruits Coping With Climate Warming
Source: Front Plant Sci. 2020 Sep 18;11:01175. doi: 10.3389/fpls.2020.01175 (PMC7536366; doi:10.3389/fpls.2020.01175)

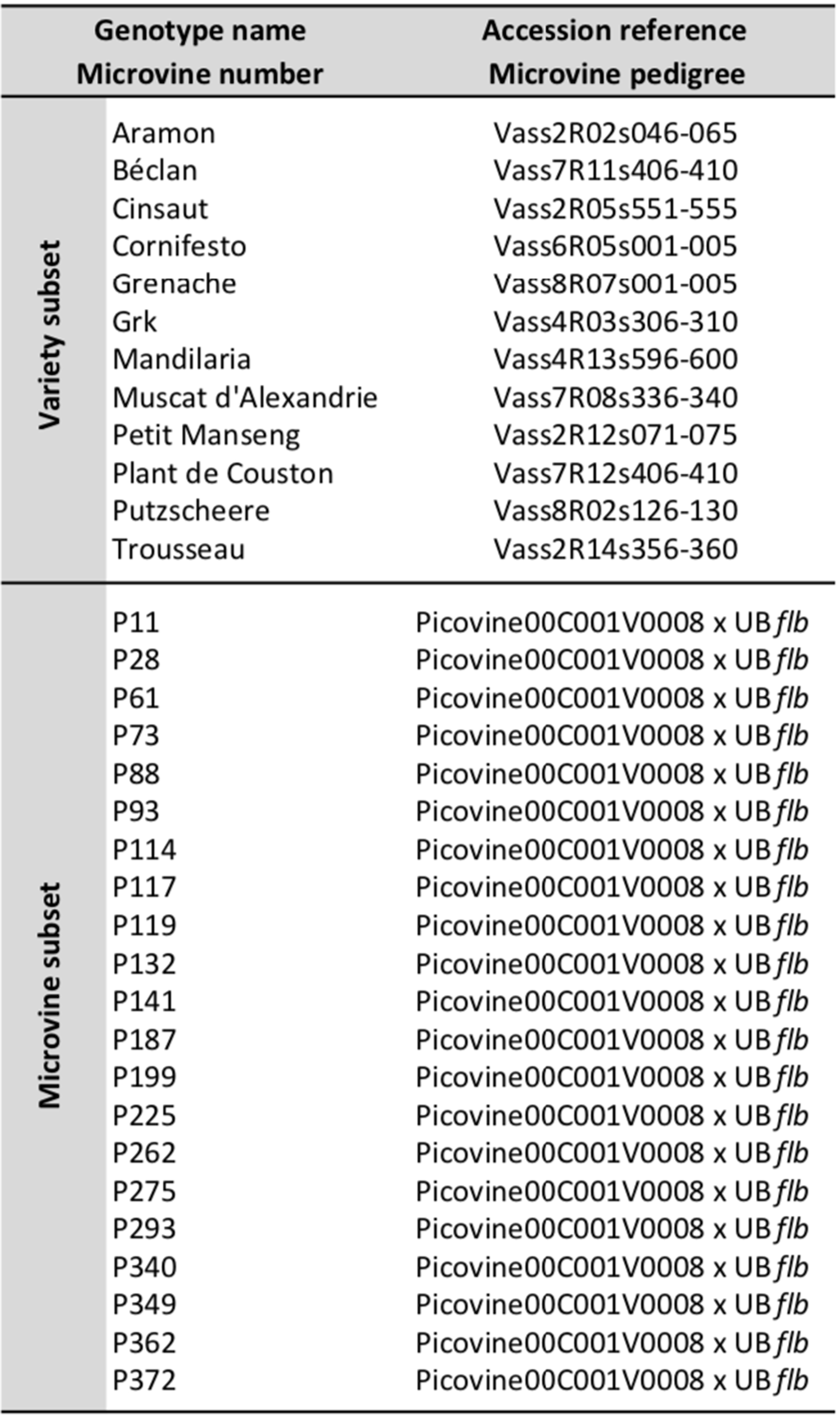

Supplement: Supplementary Table 1 — List of the genotypes of the 2 subsets of genotypes. [file Image_1.jpeg]

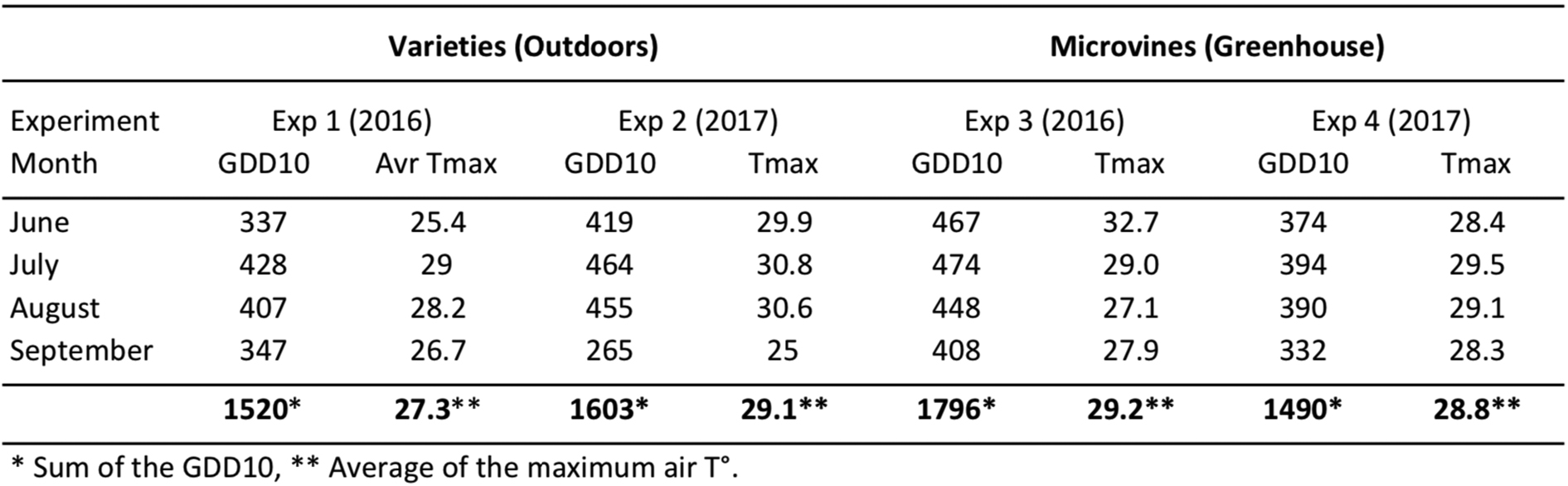

Supplement: Supplementary Table 2 — Sum of the GDD (growing degree days) in base 10 and means of the average of the maximum temperatures during the 4 months of sampling outdoors (varieties) or in greenhouse (microvines). [file Image_2.jpeg]

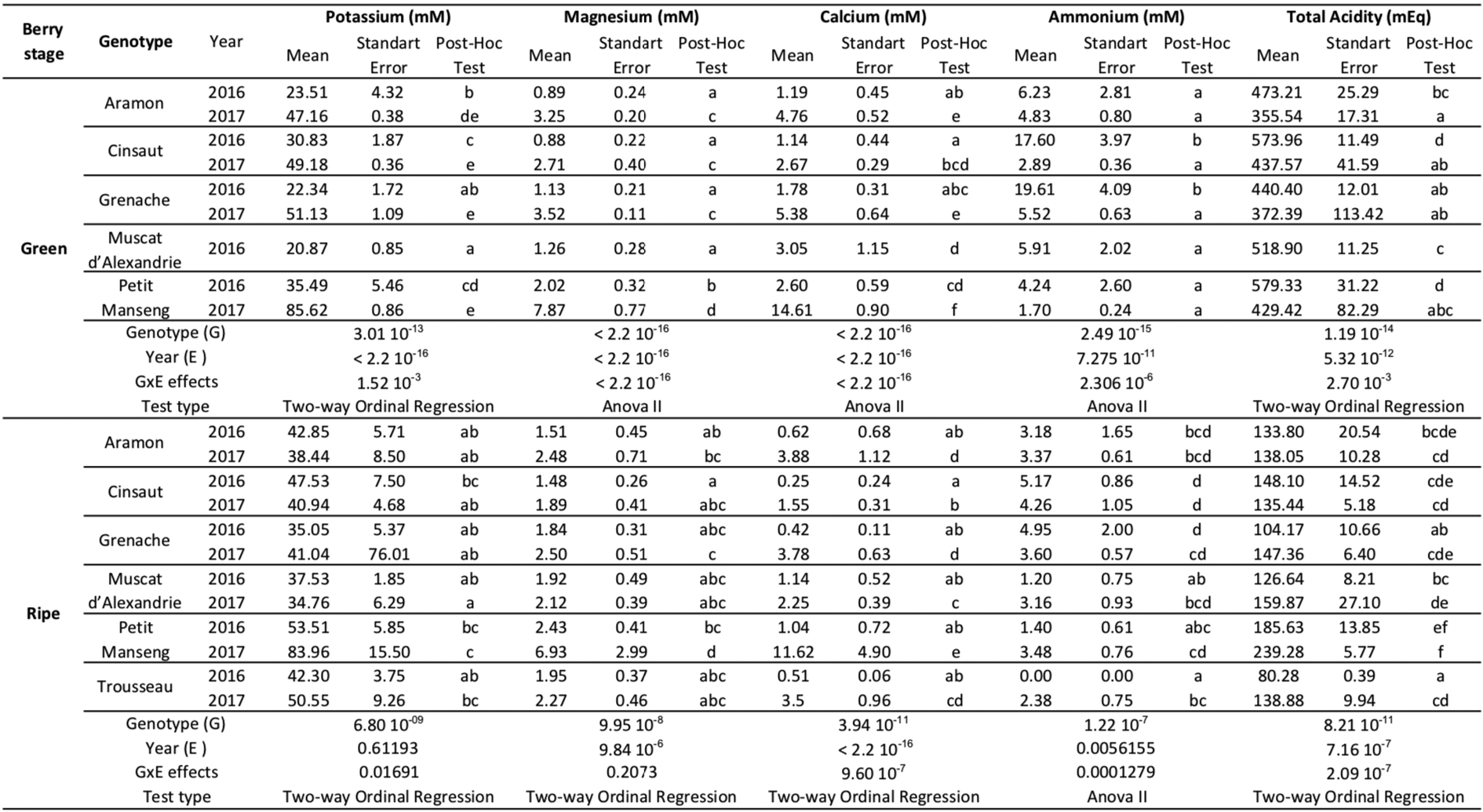

Supplement: Supplementary Table 3 — Mean values and statistics of the fruit parameters measured for the 6 V. vinifera varieties in 2016 and 2017 at green and physiological fruit ripe stage. [file Image_3.jpeg]

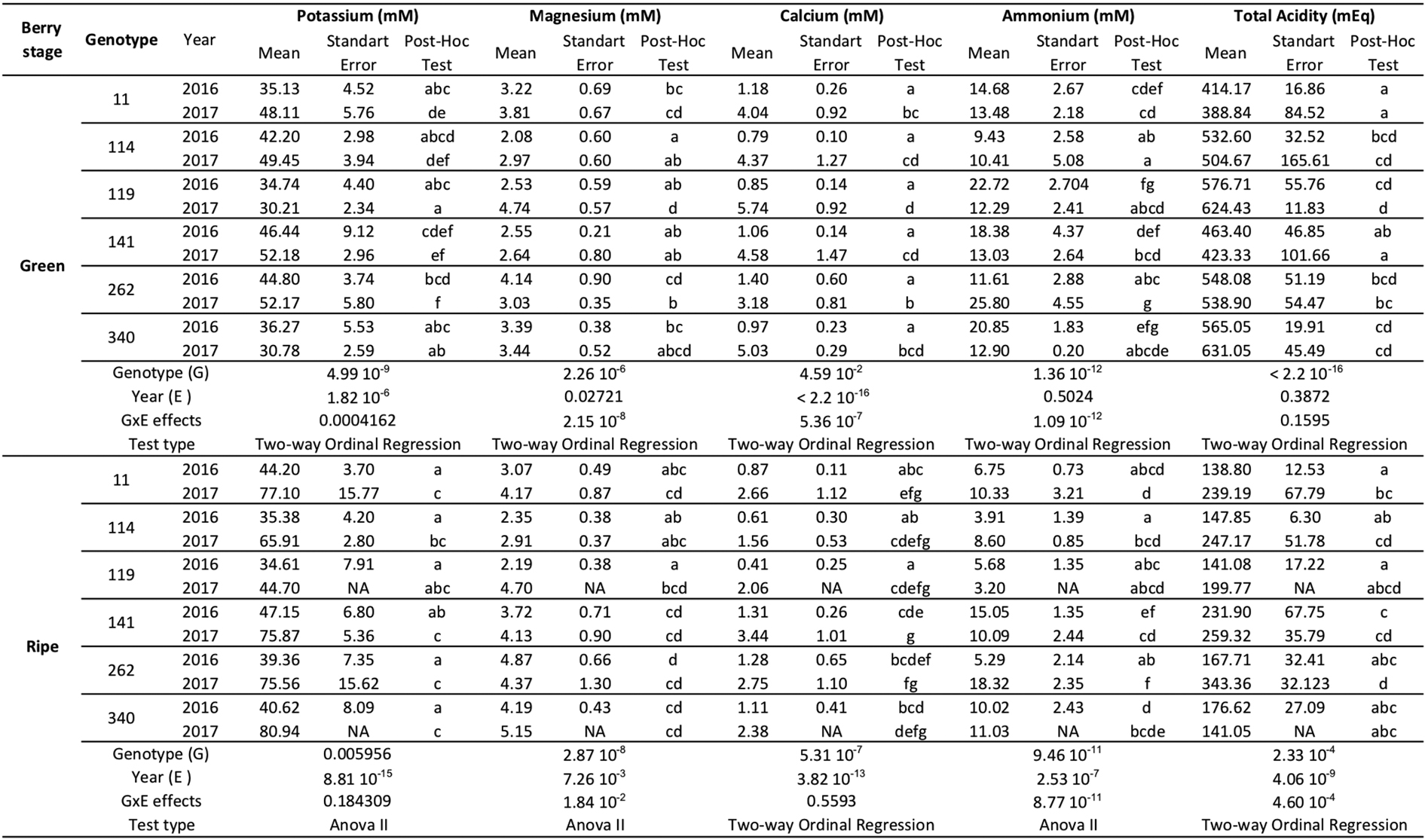

Supplement: Supplementary Table 4 — Mean values and statistics of the fruit parameters measured for the 6 V. vinifera microvines in 2016 and 2017 at green and physiological fruit ripe stage. [file Image_4.jpeg]

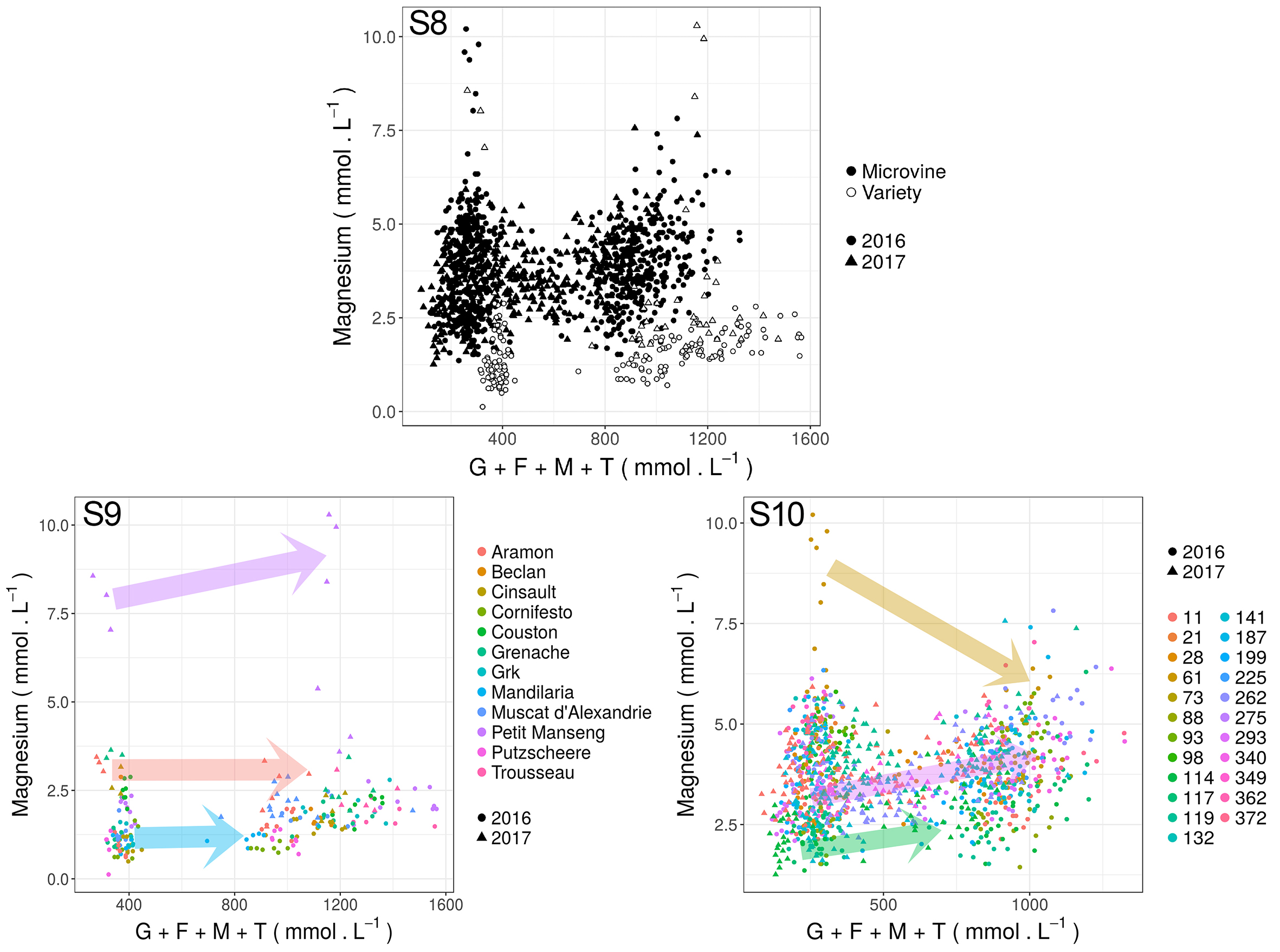

Supplement: Supplementary Figure 6 — Magnesium concentrations as a function of sum of major osmotica (glucose + fructose + malate + tartrate) during ripening for all samples of this study (S8) and in variety (S9) and microvine (S10) subsets. Colored arrows show the evolution of Mg2+ concentration for 3 genotypes of each subset. [file Image_6.jpeg]

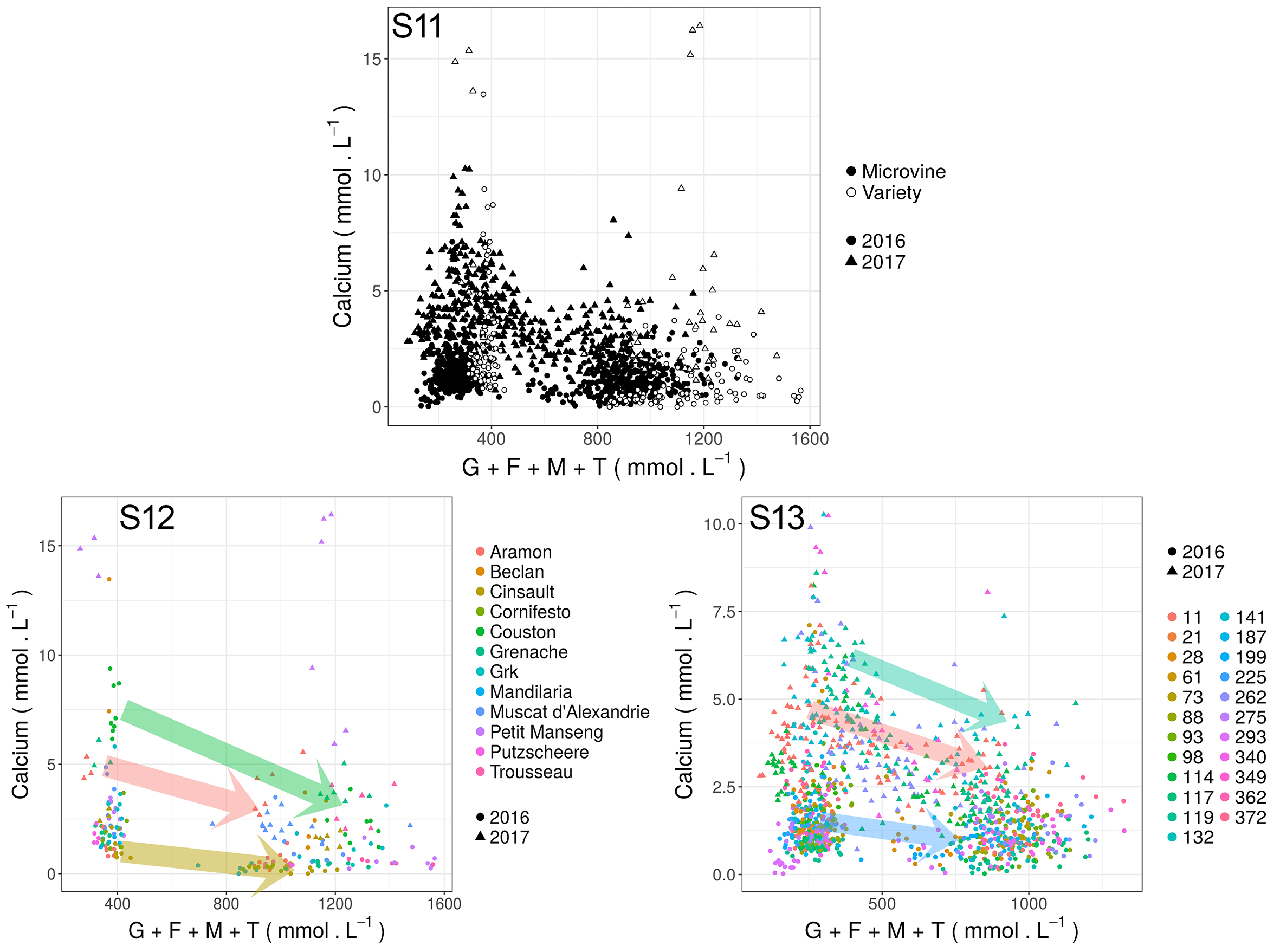

Supplement: Supplementary Figure 7 — Calcium concentrations as a function of sum of major osmotica (glucose + fructose + malate + tartrate) during ripening for all samples of this study (S11) and in variety (S12) and microvine (S13) subsets. Colored arrows show the evolution of Ca2+ concentration for 3 genotypes of each subset. [file Image_7.jpeg]

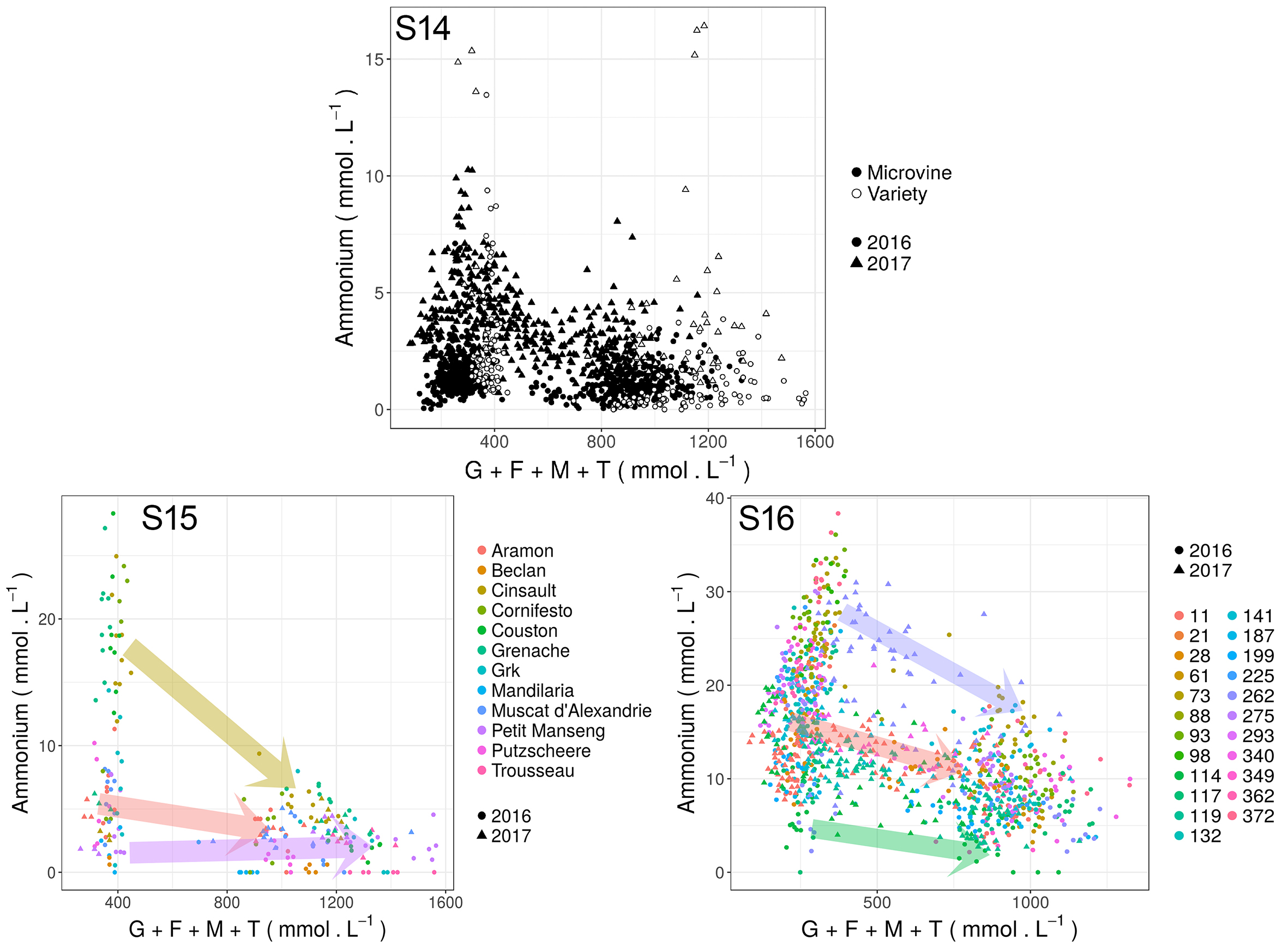

Supplement: Supplementary Figure 8 — Ammonium concentrations as a function of sum of major osmotica (glucose + fructose + malate + tartrate) during ripening for all samples of this study (S14) and in variety (S15) and microvine (S16) subsets. Colored arrows show the evolution of NH4+ concentration for 3 genotypes of each subset. [file Image_8.jpeg]

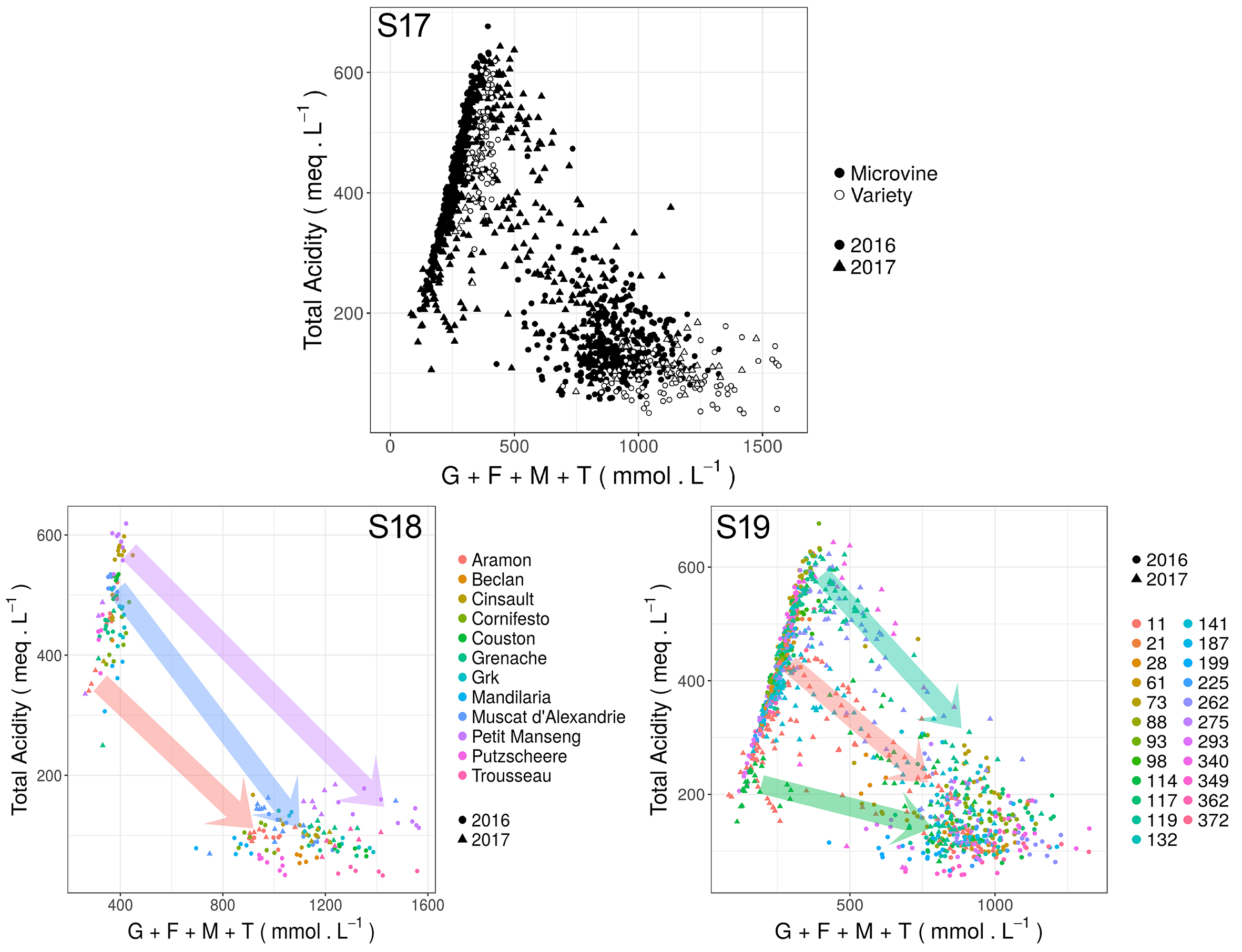

Supplement: Supplementary Figure 9 — Total acidity as a function of sum of major osmotica (glucose + fructose + malate + tartrate) during ripening for all samples of this study (S17) and in variety (S18) and microvine (S19) subsets. Colored arrows show the evolution of the total acidity for 3 genotypes of each subset. [file Image_9.jpeg]
